# Concordance of Dye-Spraying Chromoendoscopy and Virtual Chromoendoscopy for Colonic Dysplasia Detection in Longstanding Inflammatory Bowel Disease

**DOI:** 10.5152/tjg.2023.22766

**Published:** 2023-11-01

**Authors:** João Paulo Laranjeira Correia, Ana Ponte, Luisa Proença, Adélia Rodrigues, Rolando Pinho, Sónia Leite, Carlos Fernandes, Jaime Rodrigues, João Carlos Silva, Catarina Gomes, Edgar Afecto, Maria Manuela Estevinho, Pedro Mesquita, Teresa Freitas

**Affiliations:** Department of Gastroenterology, Centro Hospitalar de Vila Nova de Gaia, Espinho, Portugal

**Keywords:** Inflammatory bowel disease, dysplasia, surveillance, chromoendoscopy

## Abstract

**Background/Aims:**

In the past, dye-spraying chromoendoscopy was the technique of choice for colonic surveillance in patients with long-standing extensive inflammatory bowel disease. Recent evidence suggests that virtual chromoendoscopy is an equally acceptable technique.

**Materials and Methods:**

Eleven gastroenterologists were given a survey with 20 pairs of pictures from inflammatory bowel disease surveillance colonoscopies (10 with nondysplastic lesions, 5 with dysplastic lesions, and 5 with no lesions). Each pair contained the same image captured during colonoscopy using indigo carmine and narrow-band imaging. For each picture, the gastroenterologist assessed the presence/absence of lesion and, when a lesion was identified, assessed the presence/absence of dysplasia and delineated its margins. To compare lesion and dysplasia detection between techniques, sensitivity, specificity, and interobserver agreement were calculated. The chi-square test was used to assess the accuracy of margins delineation.

**Results:**

When assessing lesion and dysplasia detection, similar sensitivity and specificity values were obtained for both techniques. Interobserver agreement analysis revealed that dye-spraying chromoendoscopy and virtual chromoendoscopy had a moderate agreement in lesion detection but, for dysplasia detection, dye-spraying chromoendoscopy had a slight agreement [*K* = 0.11 (0.03-0.18), *P* < .01] and virtual chromoendoscopy a fair agreement [*K* = 0.30 (0.22-0.37), *P* < .01]. Margin delineation was similar between techniques.

**Conclusion:**

Sensitivity and specificity for lesion and dysplasia detection, as well as the accuracy of margins delineation, were similar between dye-spraying chromoendoscopy and virtual chromoendoscopy. Interobserver agreement for dysplasia detection was suboptimal in both techniques; however, it was superior when using virtual chromoendoscopy. These findings suggest that virtual chromoendoscopy constitutes a valid alternative for dysplasia screening in inflammatory bowel disease.

**Main Points**

- Patients with longstanding extensive inflammatory bowel disease have an increased risk of colorectal cancer.
- Dye-spraying chromoendoscopy was previously considered the gold standard of colonic surveillance in inflammatory bowel disease. The use of virtual chromoendoscopy has grown in the recent years.
- In our study, dye-spraying chromoendoscopy and virtual chromoendoscopy had similar performance in lesion and dysplasia detection, as well as in margins delineation.
- Virtual chromoendoscopy constitutes a valid alternative for dysplasia screening in inflammatory bowel disease.

## Introduction

Patients with long-standing extensive inflammatory bowel disease (IBD) have an increased risk for colorectal cancer (CRC),^1,2^ driven by cumulative inflammatory burden in the colonic mucosa.^3^ Most CRC in IBD arise from dysplastic changes in the epithelium, thus early detection of these preneoplastic lesions is a primary goal for endoscopic surveillance.^2,4^

Colonic surveillance should generally be initiated 8 years after the diagnosis in patients with ulcerative colitis (UC) with disease proximal to the rectum and in patients with Crohn’s disease (CD) affecting more than one-third of the colon.^5^

Dye-spraying chromoendoscopy (DCE), with resection or targeted biopsies of visible lesions, is a more effective technique of screening compared with standard-definition white-light (SD-WL) endoscopy with multiple nontargeted biopsies^6-8^ and until recent years was the gold standard of colonic surveillance in IBD.^5^ The use of spraying dyes highlights areas that are macroscopically elevated or depressed, friable, obscure in vasculature, and with a villous or nodular pattern in the colonic mucosa, increasing the detection rate of dysplasia.^9,10^ Previous dysplasia management guidelines considered that the evidence that supported the use of other techniques in screening patients with IBD was scarce.^1^

However, due to the advances in endoscopic technology, with the use of high-definition (HD) scopes and the emerging virtual chromoendoscopy (VCE) techniques, several studies have demonstrated that VCE is an equally high-quality technique for dysplasia and cancer screening in IBD.^11-13^ Virtual chromoendoscopy techniques are able to filter some wavelengths, underlining abnormal areas of the mucosa, similarly to DCE and without its limitations.^10^ There is also growing evidence that supports the use of HD-WL endoscopy in detecting dysplasia and neoplasia in IBD patients.^11,14^

An important point that disfavors DCE is that, as compared to VCE or HD-WL endoscopy, this technique is more time consuming.^15^

Given these considerations, the European Society of Gastrointestinal Endoscopy guidelines regarding advanced imaging for the detection and differentiation of colorectal neoplasia consider both DCE and VCE equally acceptable techniques for dysplasia surveillance in IBD.^16^

Nevertheless, the data that supports the noninferiority of HD-WL endoscopy and VCE over DCE is not always consistent.^17^

In light of these considerations, the authors aimed to evaluate lesion and dysplasia detection and the accuracy of margins delineation of lesions between DCE and VCE, among gastroenterologists from a tertiary center.

## Materials and Methods

Eleven gastroenterologists from the Gastroenterology Department of a tertiary center were given a survey containing 20 pairs of pictures from IBD surveillance colonoscopies. Each pair contained the same image captured during colonoscopy using DCE and VCE.

The 40 pictures were randomly ordered to avoid any classification bias. For each picture, the gastroenterologist assessed the presence/absence of lesion and, when a lesion was identified, assessed the presence/absence of dysplasia and delineated its margins.

The observers were instructed not to discuss the answers given and to take the survey individually. 

## Surveillance Colonoscopies

Pictures from surveillance colonoscopies, performed with HD scopes (Olympus®, series CF-H180AL, and CF-H185L) in the study center, were collected from the Gastroenterology Department database.

All colonoscopies were performed under deep sedation with propofol, administered by an anesthesiologist, at the prescribing physician’s discretion.

In all exams, the quality of the preparation was adequate (Boston Bowel Preparation Scale ≥6), and there was no active disease (Mayo subscore <2 in UC patients, simple endoscopic score <4 in CD patients). The colonoscope was advanced to the cecum, and, on withdrawal, each segment was sequentially examined for lesions. Dye-spraying chromoendoscopy was performed using 0.03% indigo carmine solution via a flushing pump.

**Figure 1.**
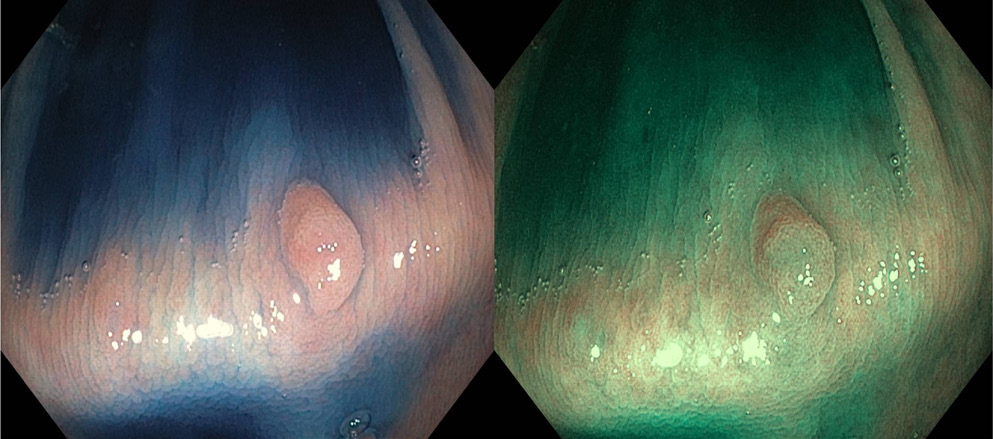
Example of a pair of pictures containing the same lesion exhibited in the survey.

Each pair contained the same image captured during colonoscopy using indigo carmine and narrow-band imaging (NBI), as shown in Figure 1.

### Image Characteristics

From the 20 pairs of images, 10 contained nondysplastic lesions (5 hyperplastic lesions, 3 nondysplastic sessile serrated lesions, and 2 inflammatory pseudopolyps), 5 contained dysplastic lesions (adenomas), and 5 contained no lesions (random pictures taken during endoscopy). Lesions characteristics are summarized in Table 1.

### Survey Organization

The 40 pictures were randomly ordered to avoid any classification bias. For each picture, the gastroenterologist had to answer 3 questions:

- Question 1: Is there any lesion in the picture? (possible answers—yes or no)
- Question 2: If there is a lesion, is it dysplastic? (possible answers—yes or no)
- Question 3: If there is a lesion, please delineate the margins.

**Figure 2.**
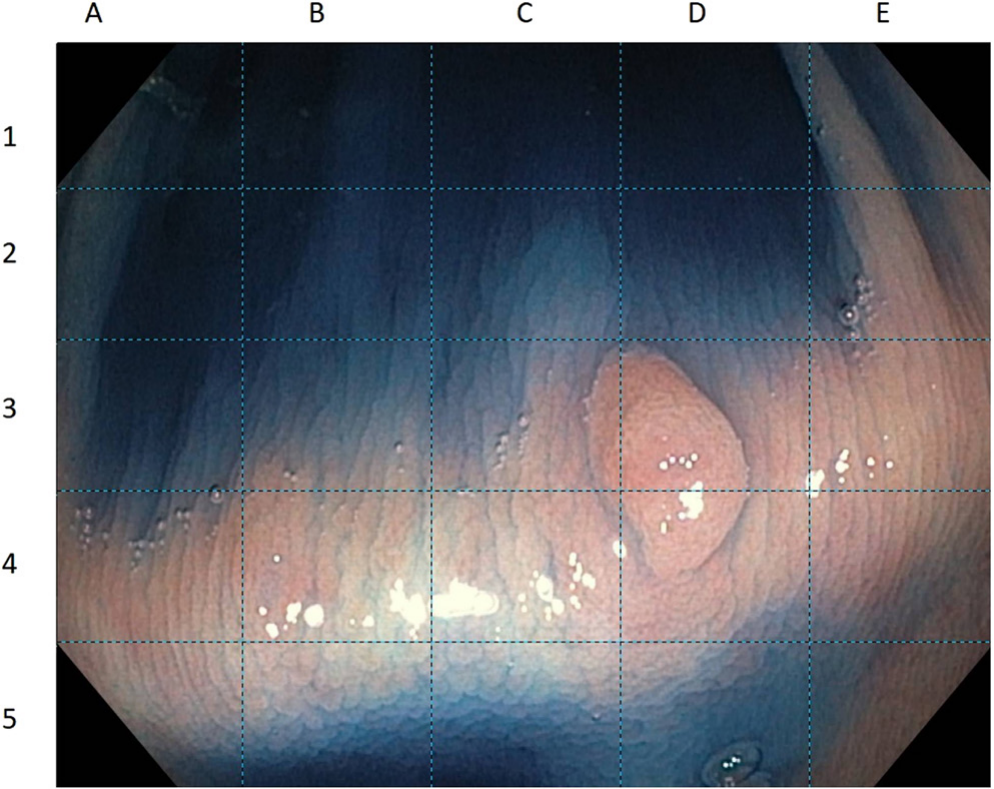
Example of a picture displayed to answer question 3 (margin delineation).

In order to answer question 3, the picture was divided into 25 identical squares. To obtain a right answer for this question, the observer had to mark correctly all the squares containing the lesion (Figure 2).

### Observers’ Degree of Experience

Concerning the use of chromoendoscopy techniques, 3 observers had low experience (Group A, endoscopists who routinely used chromoendoscopy techniques for less than 5 years) and 8 observers had high experience (Group B, endoscopists who routinely used chromoendoscopy techniques for more than 5 years). Therefore, a separate analysis of these groups was carried out to strengthen this study.

### Histology Assessment

Histological assessment was conducted by an expert gastrointestinal histopathologist. All dysplasia diagnoses were reconfirmed by a second expert gastrointestinal histopathologist.

### Statistical Analysis

Categorical variables were expressed as absolute and relative frequencies. To compare lesion and dysplasia detection between DCE and VCE, the sensitivity, specificity and inter-observer agreement (using Fleiss’ kappa (*K*) test) were obtained. Confidence intervals (CIs) for sensitivity and specificity were calculated using the Clopper–Pearson method, with a 95% degree of confidence. The authors also compared the rate of observations in which margins delineation was accurate, using the chi-square test. A separate subanalysis of Groups A and B was also conducted. The IBM Statistical Package for the Social Sciences (SPSS) version 26.0 (IBM Corp.; Armonk, NY, USA) was used for statistical analysis. A *P*-value less than .05 was considered as statistically significant.

### Ethics Committee Approval

The research was conducted ethically in accordance with the Declaration of Helsinki 2014. Approval was granted by the Ethics Committee of Centro Hospitalar de Vila Nova de Gaia/Espinho (approval number: 199/2021-(1), date: November 2021).

## Results

A total of 440 observations were taken in this survey (11 observers, 40 pictures—DCE = 220 and VCE = 220).

Regarding assessment of dysplasia detection and margin delineation, these questions could only be answered if the observer accurately identified the presence of a lesion in the picture. In 5 of the pictures using DCE and in 11 of the pictures using VCE, a lesion was present, but the observer did not identify it correctly. Therefore, for the second and the third questions, instead of 330 observations (11 observers, 30 pictures with lesions—DCE = 165 and VCE = 165), 314 observations were analyzed (DCE = 160 and VCE = 154).

When assessing lesion detection using DCE, sensitivity was 0.97 (95% CI 0.93-0.99) and specificity was 0.62 (95% CI 0.48-0.76). Interobserver agreement analysis revealed a moderate agreement for this technique—*K* = 0.58 (95% CI 0.52-0.64), *P* < .01. Regarding VCE, sensitivity and specificity for lesion detection were 0.93 (95% CI 0.88-0.97) and 0.49 (95% CI 0.35-0.63), respectively. This technique also displayed a moderate agreement among observers—*K* = 0.57 (95% CI 0.52-0.63), *P* < .01.

As for dysplasia detection using DCE, sensitivity was 0.67 (95% CI 0.53-0.79) and specificity was 0.63 (95% CI 0.52-0.72). Interobserver agreement analysis revealed a slight agreement for this technique—*K* = 0.11 (95% CI 0.03-0.18), *P* < .01. Regarding VCE, sensitivity and specificity for lesion detection were 0.74 (95% CI 0.64-0.85) and 0.60 (95% CI 0.50-0.70), respectively. However, a fair agreement among observers was obtained—*K* = 0.30 (95% CI 0.22-0.37), *P* < .01.

Concerning accuracy of margin delineation, there were also no statistically significant differences between the rate of observations with accurately defined margins delineation [DCE 124 (80.5%) vs. VCE 138 (89.6%), *P* = .17].

These results are displayed in Table 2.

### Subanalysis of Observers with Different Degrees of Experience

Regarding lesion and dysplasia detection, similar sensitivity and specificity values were obtained for both techniques, either in group A and group B (Tables 3 and 4).

Concerning accuracy of margins delineation, the authors did not find statistically significant differences between both techniques, either in group A [DCE 39 (90.7%) vs. VCE 32 (76.2%), *P* = .07] and group B [DCE 99 (83.9%) vs. VCE 92 (84.6%), *P* = .62].

## Discussion

As shown in the “Results” section, sensitivity and specificity for lesion detection and dysplasia detection, as well as the accuracy of margins delineation, were similar between DCE using indigo-carmine and VCE using NBI.

Dye-spraying chromoendoscopy and VCE were suboptimal in dysplasia detection. The distinction between dysplastic and nondysplastic lesions in IBD is still challenging, and endoscopists are often unskilled in addressing this matter. The development of structured training programs and limiting IBD surveillance colonoscopies to experienced endoscopists are 2 possible ways to overcome this issue.^18^ Endoscopists should also be more aggressive in the management of any visible lesions. *En bloc* resection in clearly demarcated lesions, without signs of deep submucosal invasion or fibrosis, should be routinely performed, over targeted biopsies, which may compromise future resection attempts. In contrast, targeted biopsies should always be performed where mucosal findings are suspicious for dysplasia or are inexplicably different from the surrounding mucosa.^19^

As for interobserver agreement in dysplasia detection, a fair agreement was found among observers when using VCE, while for DCE a slight agreement was observed. This might be related to the growing experience and the widespread use of VCE among gastroenterologists in non-IBD surveillance colonoscopies.^20^

In the past years, several studies have demonstrated that VCE is as effective as other modalities in CRC screening in IBD. González-Bernardo et al^21^ carried out a randomized controlled trial that compared DCE and VCE (iSCAN); the authors reported no differences in lesion detection and, as expected, shorter mean examination time using the first technique. Iacucci et al^11^ carried out another RCT that compared HD-WL endoscopy and VCE (using iSCAN); the authors also found no differences in lesion detection between techniques.

Concerning NBI, a randomized study with 42 patients, comparing NBI and SD-WL endoscopy, found no differences on the proportion of patients with dysplasia, but a few total lesions were found with NBI (9 vs. 12 lesions).^22^ Moreover, 2 other studies comparing HD-WL endoscopy with NBI presented also similar results: no differences in patients with dysplasia between both techniques and fewer lesions detected with NBI (5 vs. 7 and 14 vs. 16).^23,24^ Although this evidence may render NBI as not superior to SD or HD endoscopy, a more recent multicenter RCT that compared DCE with NBI found not only a similar CRC detection rate but also a shorter procedure time in the second group.^12^

However, VCE is not consistently superior to DCE throughout the literature. A recent systematic review with network meta-analysis, which compared different augmented endoscopy techniques with WL endoscopy, has shown a statistically significant superiority of DCE over WL endoscopy (odds ratio 2.12, 95% CI 1.18-5.23) in detecting dysplasia. Furthermore, DCE was associated with a higher likelihood of dysplasia detection as compared to other VCE techniques, such as NBI and I-SCAN, although without statistically significant differences.^17^

The authors present an analysis of pictures from IBD surveillance colonoscopies from a tertiary center in which VCE was not inferior to the conventionally used DCE technique, as shown by several studies assessing the detection of colitis-related neoplastic lesions.

Some authors still favor DCE over VCE.^1,25,26^ This, however, will change in the near future, due to the growing evidence of VCE noninferiority and the widespread use of endoscopic technology.

This study has several strong points. First, although this was not a real-time assessment, pictures with no lesions were included in the survey, allowing the assessment for the absence or presence of lesions. Second, although the observers had different degrees of experience, subanalysis allowed for overcoming this drawback. Third, to our knowledge, this is the first study assessing DCE and VCE with this design. Fourth, classification bias was eliminated in the survey by randomization of the pictures. Fifth, interobserver agreement analysis allowed the evaluation of the reproducibility of the technique in real-life practice.

This study has, however, some limitations. First, this study evaluates images, a quite challenging assessment, rather than a real-time assessment, which better reproduces real-life practice. Second, margin delineation was made by marking squares rather than enabling the observers to delineate the margins themselves. Third, the sample size is small compared to other studies.

In conclusion, the findings from this study suggest that VCE constitutes a valid alternative for dysplasia screening in IBD.

## Figures and Tables

**Table 1.** Lesion Characteristics

	Dysplastic (n = 5)	Nondysplastic (n = 10)
Size, median (IQR)	12 (8)	11 (8)
Paris classification – n (%)		
Type 0-Is	2 (40)	5 (50)
Type 0-IIa	2 (40)	2 (20)
Type 0-IIb	1 (20)	3 (30)

IQR, interquartile range.

**Table 2.** Sensitivity, Specificity and Interobserver Agreement of Lesion and Dysplasia Detection According to Chromoendoscopy Technique

	Dye-Spraying Chromoendoscopy				Virtual Chromoendoscopy			
	Sensitivity (95% CI)	Specificity (95% CI)	Interobserver Agreement		Sensitivity (95% CI)	Specificity (95% CI)	Interobserver Agreement	
			*K*	*P*			*K* (95% CI)	*P*
Lesion detection	0.97 (0.93-0.99)	0.62 (0.48-0.76)	0.58 (0.52-0.64)	<.01	0.93 (0.88-0.97)	0.49 (0.35-0.63)	0.57 (0.52-0.63)	<.01
Dysplasia detection	0.67 (0.53-0.79)	0.63 (0.52-0.72)	0.11 (0.03-0.18)	<.01	0.74 (0.64-0.85)	0.60 (0.50-0.70)	0.30 (0.22-0.37)	<.01

*K*, Fleiss’ kappa test.

**Table 3.** Subanalysis of Lesion Detection According to Chromoendoscopy Technique

Sensitivity (95% CI)	Specificity (95% CI)	Sensitivity (95% CI)	Specificity (95% CI)
	Dye-Spraying chromoendoscopy		Virtual Chromoendoscopy	
	Group A	0.96 (0.85-0.99)	0.60 (0.32-0.84)	0.93 (0.82-0.99)	0.40 (0.16-0.68)
Group B	0.98 (0.93-0.99)	0.63 (0.46-0.77)	0.93 (0.87-0.97)	0.53 (0.36-0.68)

**Table 4.** Subanalysis of Dysplasia Detection According to Chromoendoscopy Technique

Sensitivity (95% CI)	Specificity (95% CI)	Sensitivity (95% CI)	Specificity (95% CI)
	Dye-Spraying Chromoendoscopy		Virtual Chromoendoscopy	
	Group A	0.71 (0.42-0.92)	0.48 (0.29-0.67)	0.79 (0.49-0.95)	0.50 (0.31-0.69)
Group B	0.67 (0.56-0.78)	0.65 (0.48-0.79)	0.73 (0.56-0.85)	0.64 (0.52-0.75)
